# Influence of Solvent and Substrate on Hydrophobicity of PLA Films

**DOI:** 10.3390/polym13244289

**Published:** 2021-12-08

**Authors:** Verónica Luque-Agudo, Amparo M. Gallardo-Moreno, María Luisa González-Martín

**Affiliations:** 1 Department of Applied Physics, University of Extremadura, 06006 Badajoz, Spain; vluque@unex.es (V.L.-A.); mlglez@unex.es (M.L.G.-M.); 2 Networking Research Center on Bioengineering, Biomaterials and Nanomedicine (CIBER-BBN), 06006 Badajoz, Spain; 3 University Institute of Extremadura Sanity Research (INUBE), 06006 Badajoz, Spain

**Keywords:** polylactic acid, spatial organization, hydrophobicity, solvent-casting, substrate, solvent

## Abstract

The study of the surface properties of materials is key in determining whether the material will be suitable for medical purposes. One of these properties is hydrophobicity, which is important when assessing its behavior against bacterial adhesion. In this work, we have studied the influence of the solvent (chloroform, acetone, and tetrahydrofuran) and the substrate (glass, PTFE, silicone, and Ti6Al4V) on which polylactic acid is deposited in solution to manufacture films by solvent-casting. Thus, it has been found that there are no significant differences in hydrophobicity and surface tension among the solvents evaluated, but there are significant differences with respect to the substrates: PLA films casted on silicone are hydrophobic, while those casted on the rest of the substrates are hydrophilic. This is related to the fact that the silicone interacts with the polymer modifying its spatial arrangement, exposing its methyl groups towards the interface with the air. In this way, it has been shown that, when manufacturing PLA films, it is important to choose the right surface on which to deposit them, depending on their desired function.

## 1. Introduction

Polylactic acid (PLA) is a biopolymer widely used nowadays in various applications [1]: medical devices [2,3], prostheses [4,5], food packaging [6,7], drug delivery systems [8,9], 3D printing [10,11], supports [12], shape memory materials [13], etc. Due to its use in the biomedical field, not only the study of its mechanical and surface properties is of great importance, but also the evaluation of its ability to deal with bacterial activity and to promote cell growth and proliferation.

Regarding the antibacterial activity, one of the surface properties involved is the hydrophobicity of the material. Although the idea that bacterial adhesion is greater on hydrophobic surfaces than on hydrophilic ones is widely accepted [14], recent studies have shown that this is not always the case. Thus, Yuan et al. [15] have shown that superhydrophobic materials (water contact angle, θ_W_ > 150°) resist bacterial adhesion, and that when θ_W_ ≈ 90°, the highest level of adhesion is reached. However, this is not only applicable for superhydrophobic surfaces: in our recent study [16], we have shown that hydrophobic PLA films (θ_W_ ≈ 106°) exhibit less bacterial adhesion than more hydrophilic PLA films.

Another fact to be pointed out is the variability found in the bibliography about the water contact angle on PLA. Specifically, the reported values generally range from 65 to 80° [17,18], while slightly higher and lower values also found [16] (those cases where PLA films are prepared for superhydrophobic purposes are not taken into account). Finding a single explanation for this fact is not easy because of the number of variables acting together in the manufacture of the films, namely: preparation method (solvent-casting [19], tape-casting [20], and extrusion molding [21], generally), solvent, substrate on which the PLA solution is casted (when applicable), humidity [22], drying time and temperature, and the mechanical processes for orienting the polymer chains [23,24].

It is therefore important to determine how all these factors influence the surface properties of PLA and specifically its hydrophobicity. According to the literature, whether lower or higher θ_W_ values are obtained depends somehow on the way in which the material has been prepared, even if the composition is the same. This is interesting because, ultimately, the behavior of PLA against bacterial adhesion could be changed by simply choosing how the material is manufactured.

In our case, PLA films have been prepared by solvent-casting, varying both the solvent in which the polymer is dissolved and the substrate on which the solution is deposited. It is known that the solvent induces conformational changes in the polymer structure, which affect the air-surface interface and thus its hydrophobicity. Thus, Paragkumar et al. [25] relate conformational changes on the surface of PLLA to the migration of certain segments of the polymer chain when in contact with certain solvents, leaving the methyl groups more exposed to air. Moreover, it has also been shown that the polarity of the substrate on which the solution of polymer with some added compounds is deposited influences how the doping substances are distributed in the polymeric matrix [26], from which it is inferred that it can also affect the arrangement of the polymer chains. In this case, the authors describe how the dopant is placed at the substrate−PLA or PLA−air interface depending on the hydrophobicity of the substrate according to the interactions between the two. However, this is also applicable to undoped PLA as such interactions can occur between the hydrophobic and hydrophilic groups of the polymer chain itself with the substrate.

However, these studies usually analyze only two solvents or substrates and, in addition, focus on the three-dimensional structure and/or spatial arrangement of the polymer chains [27], with data on the contact angles of water on PLA being scarce. For this reason, the present work studies, jointly, the influence of three solvents and four substrates on the hydrophobicity of PLA films, to determine which conditions (solvent and substrate) are appropriate for the purpose of the material. The choice of green solvents would have been the preferred option due to their undoubted environmental interest [28,29]; however, the need of a good solubility of PLA and fast evaporation forced us to work with chloroform, acetone, and tetrahydrofuran (THF). Chloroform is a chlorinated solvent, acetone is a solvent belonging to the ketone group, and THF belongs to the ether group. All three are polar aprotic solvents, in which PLA is soluble. However, in acetone and THF, hydrogen bonding between molecules is more favored than in chloroform, whereas in chloroform, dispersion forces between molecules predominate. The substrates were glass, PTFE, silicone disks, and Ti6Al4V. They were selected for their hydrophobicity and/or their different nature: polymeric, metallic, or ceramic. The discussion will be also supported by the structural/compositional information of the films.

## 2. Materials and Methods

### 2.1. Material Section

Amorphous polylactic acid (PLA) was purchased from PURASORB^©^ (PDL04, Corbion, Amsterdam, the Netherlands). It is a copolymer of DL-lactide with an inherent viscosity midpoint of 0.4 dL g^−^^1^ and was used as received. Crystalline PLA (PLA2003D) was purchased from NatureWorks LLC (Blair, NE, USA) and was used as received.

Chloroform (Sigma-Aldrich, Merck, Madrid, Spain, purity > 99.5%) and acetone (PanReac AppliChem, Barcelona, Spain, technical grade) were used without further purification. Tetrahydrofuran (THF) was purchased from PanReac AppliChem (Barcelona, Spain, purity > 99.9%) and was stored with metallic sodium wires to prevent humidity.

### 2.2. Substrates Preparation

Polytetrafluoroethylene (PTFE, Lork Industrias, Barcelona, Spain) and glass (Garvaglass S.L.L., Santa Perpetua de Mogoda, Barcelona, Spain) samples were immersed in chromic acid for 15 min and then rinsed with distilled water. They were dried in an oven and stored in a desiccator until use. Ti6Al4V discs (DKSH, Zurich, Switzerland) were mechanically polished to mirror finish and cleaned and then treated to achieve a passivation layer on their surface: the discs were immersed in distilled water and kept in an oven for 72 h. After that time, they were left to dry and stored in the desiccator until use. Silicone discs (de Buyer, Vosges, France) were rinsed with absolute ethanol and dried with a flow of N_2_. They were kept in the desiccator until use. All samples have a diameter of 25 mm.

### 2.3. Films Preparation

For amorphous PLA films, polylactic acid (PDL04) particles were dissolved in chloroform, acetone, and tetrahydrofuran (20% w v^−1^) using a rotator stirrer (JP Selecta, Barcelona, Spain). Then, 0.4 mL of each mixture was placed on the different substrates, and samples were left to dry first at room temperature for 24 h, and later in an oven at 70 °C for 24 h to completely remove any remaining solvent.

For crystalline PLA films, our previously reported method was followed [16]. In brief, polylactic acid (PLA) particles (PLA2003D) were dissolved in chloroform (5% w v^−1^) using a rotator stirrer (JP Selecta, Barcelona, Spain). Then, 1 mL of each mixture was placed on the different substrates, and samples were left to dry first at room temperature for 24 h, and later in an oven at 70 °C for 24 h, to completely remove any remaining solvent.

Samples are denoted as PLA-C for those prepared in chloroform, PLA-A for those prepared in acetone, and PLA-T for those prepared in THF.

### 2.4. Contact Angle Measurements and Surface Tension Calculations

The measurements were carried out with a Krüss goniometer (Krüss, Hamburg, Germany) by the sessile drop method and using the Drop Shape Analyzer software (Version 1.12.2.06901, Krüss GmbH, Hamburg, Germany). The values of contact angle are the average of at least fifteen drops deposited on three different samples and reported with the standard deviation. In the experiment, three liquids were used: deionized water, formamide (Fluka), and diiodomethane (Fluka).

The surface tension components of the films were calculated according to the approach of van Oss et al. [30,31,32], and uncertainties were calculated using error propagation.

### 2.5. X-ray Diffraction

XRD was performed using a Bruker D8 Advance (Rheinstetten, Germany), with Bragg–Brentano geometry and CuK α_1_ radiation (λ = 1.5406 Å).

### 2.6. AFM

Morphology of surface of PLA-C films casted on PTFE, glass, and silicone disks was evaluated by using an atomic force microscope (AFM) (Agilent AFM 5500, Agilent Technologies, California, CA, USA) operating at room temperature. Rectangular silicon cantilevers used have a nominal spring constant of 0.2 N m^−1^ (ESPA-V2/Sb doped Si, Bruker, Billerica, MA, USA).

### 2.7. ToF-SIMS

Time of flight secondary ion mass spectrometry (ToF-SIMS) analyses of samples were performed with a Tof-SIMS^5^ (ION TOF, Münster, Germany) using a Bi_3_^2+^ as primary gun, which operated at 25 keV. The total ion dose used to acquire each spectrum was above 10^12^ ions cm^−2^. Negative spectra were recorded, and a pulsed low-energy electron flood gun was used for charge neutralization.

### 2.8. XPS

X-ray photoelectron spectroscopy was carried out on a UPS-XPS Multilab System 2000 Thermo Fisher Scientific spectrometer. A monochromated Mg K α X-ray source was used at a nominal power of 400 W to record spectra at normal emission. Survey spectra were captured using a pass energy of 15 eV, and core levels were captured at 15 eV.

## 3. Results and Discussion

The solvent in which the PLA is solved and the substrate on which the mixture is deposited can influence the conformational structure adopted by the polymer, either by interactions between the solvent and the PLA or between the polymer and the functional groups present on the surface of the substrate. These possible conformational changes can be evaluated by measuring the hydrophobicity of the films and even their surface tension.

The contact angles of water, formamide, and diiodomethane of the films prepared on the four substrates and in three different solvents were measured (Table 1 and Table S1). Previous tests were carried out measuring both the side of the film exposed to air and the side in contact with the substrate, obtaining the same values in both cases, so that only those corresponding to the side of the film exposed to air are shown.

As it can be inferred from the table, data can be classified in two sets: one concerning silicone and another concerning glass, PTFE, and titanium alloy. Whatever the solvent used, PLA films prepared on silicone have a water contact angle about 30 degrees higher than those casted on another surface. This means that the behavior of PLA switches from hydrophilic to hydrophobic simply by depositing it on another material. This dramatic difference is maintained in the formamide contact angle, which also shows an increase of about 30 degrees. However, when the behavior of a non-polar liquid, such as diiodomethane, is evaluated, hardly any difference is found, so the interaction between the polymer and the substrate must be of the polar type and extends through the entire thickness of the film; hence, differences are found when measuring contact angles on the side exposed to the air. For films casted on silicone, glass, and Ti6Al4V, no differences are observed in the hydrophobicity of PLA depending on the solvent in which it is dissolved, which suggests that the influence of the substrate is much greater than that of the solvent, if any.

On the contrary, in the particular case of films deposited on PTFE, differences in the contact angles of water and formamide are observed when the polymer solution is made up in acetone compared to chloroform and THF, indicating that the polymer−solvent interaction could be greater than the polymer−substrate one. This fact is in accordance with what was previously established by Ringard-Lefebvre and Baszkin [33]. These authors found that the properties of the air−water interface in PLA monolayers are strongly dependent on the properties of the dispersion solvent, as the solvent induces changes in the macromolecular spatial arrangement of the polymer. They also studied chloroform, acetone, and THF, arguing that the latter two behave in one way and the former in another: in chloroform, the methyl groups were oriented towards the air, making the films more hydrophobic, whereas in acetone a more folded structure predominated.

To test whether the differences in hydrophobicity of PLA films depending on the substrate are due to differences in the topography and roughness of the samples, the samples were analyzed by AFM. Thus, in PLA-C films deposited on PTFE, glass, and silicone, it was observed that, in all three cases, the topography was very similar. All three films are very smooth, with no well-defined or distinct structure on any of them. The measured average roughness (Sa) is as follows: 0.25 ± 0.20 nm, 0.23 ± 0.13 nm, and 0.18 ± 0.03 nm for PTFE, silicone, and glass, respectively. In view of these results, we could not associate changes in hydrophobicity with changes in the topography of the samples. 

**Figure 1:**
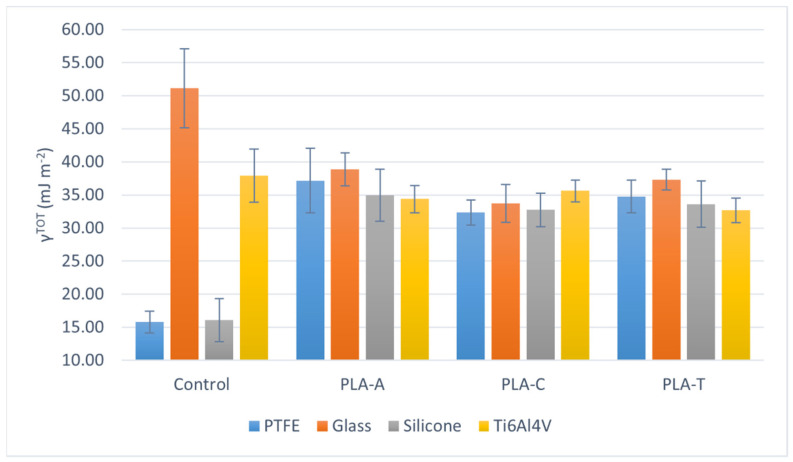
Total surface tension (γ^TOT^) depending on the solvent, for each casting substrate.

**Figure 2:**
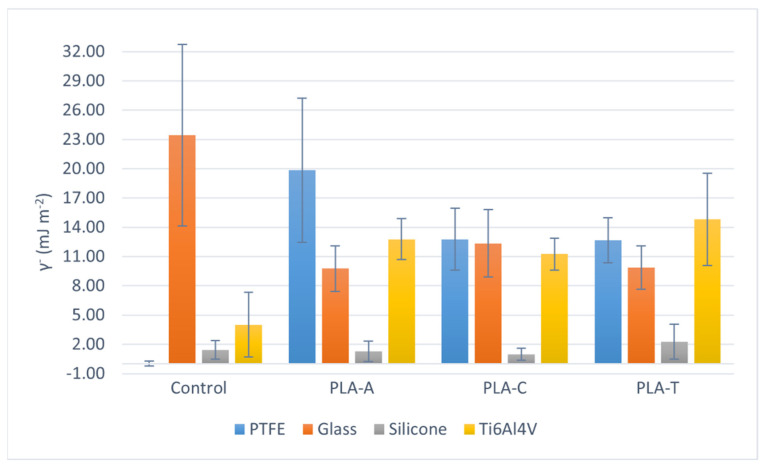
Electron donor parameter (γ−) depending on the solvent, for each casting substrate.

**Figure 3:**
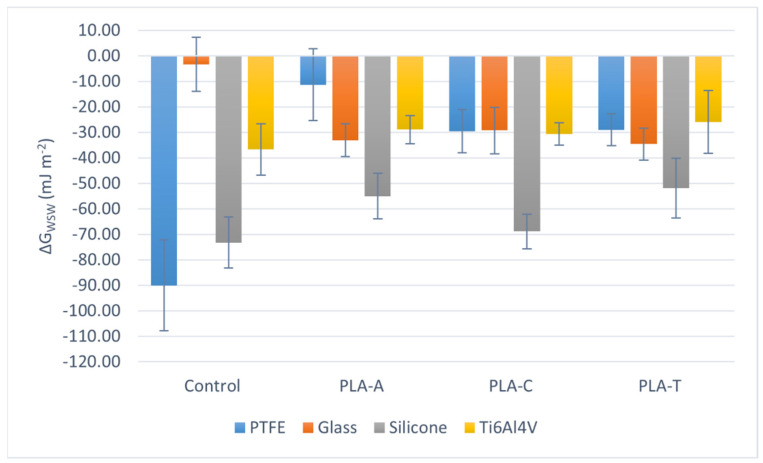
ΔG_SWS_ depending on the solvent, for each casting substrate.

Differences in contact angles translate into changes in the surface tension of the films. Table S2 in the Supporting Information lists all the surface tension components calculated according to the van Oss model [30,31,32]. Figure 1, Figure 2 and Figure 3 graphically represent the surface tension components in which the most relevant changes are found as a function of the solvent for each substrate. The total surface tension (γ^TOT^, Figure 1) of the hydrophobic surfaces is 2 or even 3 times lower than that of glass or Ti6Al4V. However, the surface tension of PLA films casted on them reach very similar values, comprised between 30 and 40 mJ m^−2^. Since the total surface tension is the sum of the acid−base (γ^AB^) and Lishitz−van der Waals (γ^LW^) components, it is possible to discern whether the interactions are polar or non-polar, respectively. 

According to the data in Table S2, the trend of γ^LW^ is the same as that of γ^TOT^, so that the changes occur in γ^AB^, and in particular, in the electron-donor parameter of the acid−base component of surface tension (γ^−^, Figure 2). This polar parameter is very high for the glass surface compared to the other three substrates. However, in PLA films casted on glass, PTFE, and Ti6Al4V, γ^−^ tends to equalize (considering the uncertainties): these values decrease in samples prepared on glass and increase in PTFE and titanium alloy. It is noteworthy that PLA deposited on silicone has a very low γ^−^ value, very similar to that of the starting surface, in contrast to the γ^−^ value of PLA casted on PTFE, which is also hydrophobic. Thus, although both are hydrophobic surfaces, their behavior with respect to electron transfer and electron acceptance is very different. Mi et al., in their paper on the application of N_2_ plasma to polyacrylamide, established that when γ^−^ is much larger than γ^+^, the polymer behaves as a Lewis base, i.e., it has the ability to donate an electron pair when confronted with a Lewis acid [34]. Thus, in view of the results, it can be said that PLA films deposited on glass, PTFE, and titanium alloy behave as a Lewis base. In the case of PLA casted on silicone, the values of γ− and γ^+^ are low and very similar, so that it can act as both a weak acid and a weak Lewis base, depending on the system it is facing. Furthermore, this trend is observed regardless of the solvent in which the polymer is prepared.

This behavior is supported by examining the interaction free energy of two surfaces immersed in water (ΔG_SWS_). The more negative this value is, the more the surfaces interact with each other and repel water. This is ultimately another measure of the hydrophobicity of the system. Thus, Figure 3 shows that the starting surfaces are very hydrophobic (PTFE and silicone), hydrophilic (glass), or moderately hydrophilic (Ti6Al4V). However, all PLA films show an ΔG_SWS_ value around −30 mJ m^−2^, except for those casted on silicone, whose average values are between −50 and −70 mJ m^−2^.

When comparing the total surface tension of PLA films prepared in the three solvents, it is observed (Figure 1) that there are no significant differences between the values of PLA-A, PLA-C, and PLA-T. The values range between 35 and 40 mJ m^−2^ for PLA-A, and between 30 and 35 mJ m^−2^ for PLA-C and PLA-T, for all substrates. In the case of the electron donor parameter, taking into account the uncertainty of Figure 2, there are no significant differences between the solvents, and the same occurs when analyzing ΔGSWS (Figure 3): the mean value in the case of PLA-A deposited on PTFE seems to be higher than the others (PLA-C and PLA-T), but this is meaningless due to the large uncertainty.

Since no significant differences are found in terms of the use of different solvents, but only in relation to the substrate on which the films are deposited and, in particular, between silicone and other surfaces, we will focus on these substrates from now on, for PLA-C films.

**Figure 4:**
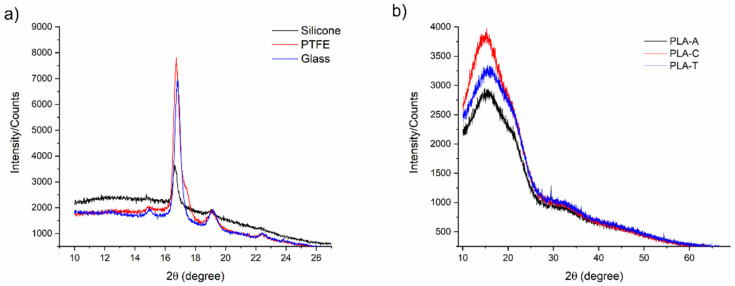
(**a**) XRD diffractograms of PLA-C films casted on PTFE, glass, and silicone. (**b**) XRD diffractograms of PLA-C, PLA-A, and PLA-T films casted on silicone.

One of the possible consequences of PLA−substrate interaction is the change in the crystalline structure and/or spatial arrangement of the polymer when the film is formed. Since the PLA used to manufacture the films is amorphous and is difficult to elucidate whether there have been changes in the spatial arrangement of the polymer, crystalline PLA films have been prepared to determine if PLA crystallizes in different structures depending on the substrate. X-ray diffractograms were recorded (Figure 4) after checking that the hydrophobicity of crystalline PLA-C films was analogous to that of amorphous PLA films (θ_W_ were as follows: 72° ± 2°, 73° ± 2°, 73° ± 1°, and 106° ± 2° for PTFE, glass, Ti6Al4V, and silicone, respectively). PLA is known to crystallize into three main structures: α, β, and δ (or α’), which can be considered a distortion of the α phase, although it is a distinct crystalline phase [35,36,37]. In the α phase, the methyl groups of the polymer chain are oriented longitudinally outwards from the helix. In the β phase, although the methyl groups also face outwards, the chains take on a globular conformation, so that not as many methyl groups are exposed towards the surface compared to the α phase. 

The peaks found in the diffractograms of PLA-C deposited on glass and PTFE correspond unequivocally to the α crystalline phase, the characteristic peaks of which are located at 2θ = 14.8°, 16.7°, 19.1°, and 22.4°. In contrast, Figure 4a shows how the characteristic peaks of PLA prepared on silicone are slightly shifted to the left, with 2θ lower values. This crystalline form is not the β phase, as the characteristic peaks at 2θ = 25.8°, 26.3°, and 28.1° do not appear in the diffractogram, nor the δ phase, as neither is found the peak at 2θ = 24.5°. It could therefore be the same α phase, but distorted, without constituting a distinct crystalline phase. This again supports the theory that the silicone somehow interacts with the polymer during the drying process and is responsible for its peculiar behavior. Narladkar et al. [27] studied how PLA macromolecules, solved in dichloromethane, were ordered depending on the substrate, namely mica and silicon wafer, and determined that PLA deposited on mica acquired an elongated structure, being that the films are hydrophobic because the methyl groups were oriented outwards. PLA casted on silicon wafer acquires a globular or coil structure. However, the results obtained in terms of hydrophobicity seem to contradict this theory since, in our case, PLA is more hydrophobic when casted on silicone, compared to other hydrophobic and hydrophilic surfaces. Moreover, this interaction is independent of the solvent in which the PLA is dissolved, as shown in Figure 4b, which shows the diffractograms of PLA-C, PLA-A, and PLA-T films casted on silicone, with no differences between them.

Since the crystal structure does not vary according to the substrate on which the polymer is deposited, it is plausible to think that what does change is the spatial arrangement of the polymeric chains: how they are arranged and the spacing between them, either due to PLA−substrate interactions or steric hindrance. Amorphous PLA-C films casted on glass, PTFE, and silicone were analyzed by ToF-SIMS to calculate the ratio of hydrophobic to hydrophilic groups on the surface: I_CH3_/I_CH3+O_. Hydrophobic groups are the methyl groups of the polymer chain (I_CH3_), while hydrophilic groups are those low molecular weight oxygen-containing species (I_O_, being O: O^−^, OH^−^, O_2_^−^, CHO_2_^−^, CO^−^, and CHO^−^). The results are as follows: 0.0151 ± 0.0003, 0.0273 ± 0.0035, and 0.0141 ± 0.0006, for PTFE, silicone, and glass, respectively. Thus, it is observed that in the case of silicone, there are more methyl groups on the surface; in fact, it is almost twice as much compared to glass and PTFE. This indicates that there are more hydrophobic species on the surface of films deposited on silicone, which could explain its hydrophobicity. Moreover, in view of these results, it can be attributed to the presence of oxygen-containing groups that PLA deposited on glass, PTFE, and titanium alloy behaves as Lewis bases, as previously stated.

**Figure 5:**
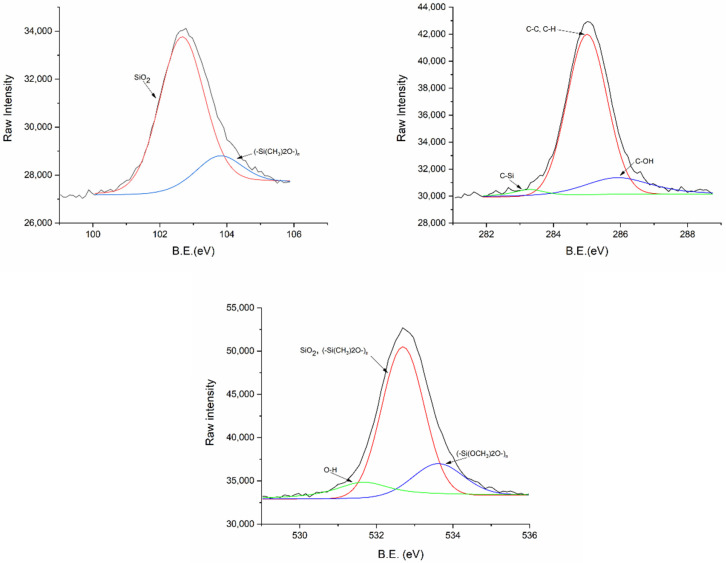
XPS high-resolution spectra of Si2p, C1s, and O1s for silicone substrate. The arrows assign each bond in each of the curves obtained after the deconvolution of the main peak.

At this point, since the substrate that shows very different results compared to the rest is silicone, it is important to know its chemical nature in order to propose a mechanism of interaction between the polymer and the substrate. The surface chemistry of PTFE, glass, and Ti6Al4V is widely known [38,39,40], but not that of the silicone used in this work, so we analyzed it by XPS (Figure 5).

The deconvolution of the core levels for the silicone O1s, C1s, and Si2p shows three peaks for O1s and C1s and two peaks for Si2p. Thus, for the latter, the peaks corresponding to the Si-O_2_ and (-Si(CH_3_)_2_O-)_n_ bonds are found at 103.80 and 102.65 eV, respectively. The peak assignments of the C1s peaks are C-C, C-H (285.00 eV), C-OH (285.90 eV), and C-Si (283.30 eV). Finally, the O1s peak is due to contributions from Si-O_2_ (532.70 eV), (-Si(CH_3_)_2_O-)_n_ (533.61 eV), and O-H (531.66 eV) bonds. Based on these results, it appears that the silicone substrate consists mainly of polydimethylsiloxane.

PTFE consists of repeating -CF_2_- units, and although one might think that the F atoms would make it a very reactive molecule, it is not, as the F atoms are strongly involved in intermolecular bonds with C atoms of neighboring chains, so it does not interact with PLA. In the cases of glass and Ti6Al4V, Si-OH and Ti-OH groups are found, respectively, which can interact via hydrogen bonds with the hydrophilic groups of the PLA. Since the helical structure of the α crystalline phase is clear, it would imply that the methyl groups would be inward facing, thus explaining the low/no hydrophobicity of PLA.

Finally, the case of silicone is singular, because, although it has Si-OH groups on its surface, it also has methyl groups, so that, although there could be hydrogen bonds interactions, there is also steric hindrance between these groups and the methyl groups of PLA. This could cause the crystalline structure to show disruptions, so that there are regions where the methyl groups are facing outwards and others where they are facing inwards, and even twists within the crystalline structure itself, which would account for the X-ray diffractogram obtained for these films. This also explains the lower crystallinity of PLA-C films casted on silicone and, similarly, this argument can be extrapolated to amorphous PLA, except that instead of crystalline structures, there are different spatial arrangements in which the methyl groups of the polymer chain are more or less exposed towards the interface with the air. This suggests that the conformational changes of the polymer chain depend on the chemistry of the substrates and not on their hydrophobic or hydrophilic character. For this reason, different results are obtained when comparing PTFE and silicone.

Moreover, since the singularity appears in silicone, it is important to evaluate whether the PLA−substrate interaction occurs independently of the thickness of the film. For this reason, crystalline PLA films of different thicknesses were casted on and peeled off the silicone, and the water contact angles were measured (Table 2). The results confirmed that the interaction extends over the entire film thickness (based on the measured angles), considering that the experimental uncertainties do not show significant differences.

Therefore, the PLA−silicone interaction is so strong that it determines the spatial arrangement of the polymer chains throughout the thickness of the film and is even independent of the solvent in which the polymer has been dissolved. In this way, it would be possible to modify the hydrophobicity of PLA films by choosing the substrate on which these films are to be deposited.

## 4. Conclusions

Regarding the influence of the PLA solvent on the hydrophobicity of the films prepared by solvent-casting, no significant differences were found between chloroform, acetone, and THF. Neither do they seem to influence the crystalline structure/spatial arrangement of the polymer chains.

However, it has been shown that the substrate on which different PLA solutions are deposited to manufacture films influences the hydrophobicity of the film, with an increase of 30° in the water contact angle when it is casted on silicone, compared to when it is casted on glass, PTFE, or Ti6Al4V, probably due to chemical interactions between the silicone and the polymer. This change could be associated with the higher presence of methyl groups on the surface, as deduced from calculations based on the relative intensities of functional groups obtained by ToF-SIMS. The chemical composition of the silicone surface revealed by XPS supports this theory.

According to our results, it seems that PLA−substrate interaction predominates over PLA−solvent interaction.

In conclusion, the surface on which PLA films are prepared could also be considered as another method of changing some of its surface properties.

## Figures and Tables

**Table 1:** Contact angles of water (θ_W_), formamide (θ_F_), and diiodomethane (θ_D_) of PLA films casted on PTFE, glass, Ti6Al4V, and silicone, according to the solvent.

	θ_W_ ± s_W_ [°]				θ_F_ ± s_F_ [°]				θ_D_ ± s_D_ [°]			
	Control	PLA-C	PLA-A	PLA-T	Control	PLA-C	PLA-A	PLA-T	Control	PLA-C	PLA-A	PLA-T
PTFE	113 ± 5	76 ± 3	64 ± 5	74 ± 2	92 ± 4	64 ± 2	54 ± 5	60 ± 3	83 ± 4	48 ± 2	47 ± 5	46 ± 3
Glass	43 ± 4	74 ± 4	71 ± 2	74 ± 2	24 ± 9	62 ± 3	52 ± 4	56 ± 2	48 ± 1	49 ± 3	45 ± 3	46 ± 2
Ti6Al4V	74 ± 5	76 ± 4	74 ± 2	74 ± 2	43 ± 1	66 ± 2	61 ± 2	59 ± 3	54 ± 3	44 ± 1	47 ± 3	47 ± 3
Silicone	113 ± 2	106 ± 2	111 ± 2	108 ± 2	100 ± 2	88 ± 3	95 ± 4	93 ± 7	82 ± 6	53 ± 4	53 ± 6	55 ± 5

**Table 2:** Contact angles of water (θ_W_) of PLA films prepared in chloroform and casted on silicone according to their thickness. Thickness is measured at the center of the film.

Film Thickness (µm)	θ_W_ ± s_W_ [°]
26	104 ± 4
27	106 ± 2
58	106 ± 2
273	104 ± 3
279	101 ± 2
294	96 ± 4
302	107 ± 4
317	94 ± 5
335	102 ± 5
402	103 ± 2

## Data Availability

Data are contained within the article or Supplementary Materials.

## Supplementary Materials

The following are available online at https://www.mdpi.com/article/10.3390/polym13244289/s1, Table S1: Contact angles of water (θ_W_), formamide (θ_F_), and diiodomethane (θ_D_) of PTFE, glass, Ti6Al4V, and silicone, Table S2: Surface free energy (γ^TOT^), components (γ^LW^: Lifshitz–van der Waals, γ^AB^: acid–base), and surface free energy of interaction of surfaces immersed in water (ΔG_SWS_) of PLA films depending on the solvent, for each casting substrate. All values are expressed in mJ m^−2^.

## Sample Availability

Samples are not available.
