# Supplementary material for: Influence of Solvent and Substrate on Hydrophobicity of PLA Films
Source: Polymers (Basel). 2021 Dec 8;13(24):4289. doi: 10.3390/polym13244289 (PMC8707572; doi:10.3390/polym13244289)
Supplement: Supplementary file 1 [file polymers-13-04289-s001.zip › polymers-1494153-supplementary.pdf]

Influence of Solvent and Substrate on Hydrophobicity of PLA Films

Verónica Luque-Agudo [1,2,3], Amparo M. Gallardo-Moreno [1,2,3,*] and M. Luisa González-Martín [1,2,3]

[1] University of Extremadura, Department of Applied Physics, Badajoz, Spain.
[2] Networking Research Center on Bioengineering, Biomaterials and Nanomedicine (CIBER-BBN), Badajoz, Spain.
[3] University Institute of Extremadura Sanity Research (INUBE), Badajoz, Spain.
* Correspondence: amparogm@unex.es

SUPPORTING INFORMATION

Table S1. Contact angles of water (θ_W), formamide (θ_F) and diiodomethane (θ_D) of PTFE, glass, Ti6Al4V and silicone.

	$\theta_W \pm s_W$ [º]	$\theta_F \pm s_F$ [º]	$\theta_D \pm s_D$ [º]
**PTFE**	113 ± 5	92 ± 4	83 ± 4
**Glass**	43 ± 4	24 ± 9	48 ± 1
**Ti6Al4V**	74 ± 5	43 ± 1	54 ± 3
**Silicone**	113 ± 2	100 ± 2	82 ± 6

Table S2. Surface free energy (γ^{TOT}), components (γ^{LW}: Lifshitz–van der Waals, γ^{AB}: acid–base) and surface free energy of interaction of surfaces immersed in water (ΔG_{SWS}), of PLA films depending on the solvent, for each casting-substrate. All values are expressed in mJ m^{-2}.

**Substrate**	**Sample**	γ^{TOT}	**σ** γ_{TOT}	γ^{LW}	**σ** γ_{LW}	γ^{AB}	**σ** γ_{AB}	γ^{+}	**σ** γ_{+}	γ^{-}	**σ** γ_{-}	ΔG_{SWS}	**σ** ΔG_{SWS}
PTFE	Control	15.77	1.66	15.62	1.40	0.15	0.90	0.26	0.40	0.02	0.27	-90.03	17.86
	PLA-A	37.18	4.88	29.86	2.62	7.32	4.12	0.68	0.72	19.85	7.39	-11.32	14.09
	PLA-C	32.36	1.90	30.33	1.20	2.03	1.47	0.08	0.12	12.77	3.17	-29.55	8.54
	PLA-T	34.77	2.48	31.34	1.43	3.43	2.03	0.23	0.27	12.68	2.31	-28.94	6.18
Glass	Control	51.13	5.96	28.94	1.25	22.19	5.83	5.26	1.81	23.42	9.31	-3.33	10.63
	PLA-A	38.86	2.48	32.42	1.42	6.44	2.03	1.06	0.62	9.75	2.32	-33.07	6.43
	PLA-C	33.70	2.85	30.16	1.83	3.54	2.18	0.25	0.30	12.35	3.45	-29.28	9.14
	PLA-T	37.31	1.58	32.37	0.82	4.94	1.35	0.62	0.31	9.86	2.21	-34.64	6.20
Silicone	Control	16.10	3.26	15.18	3.08	0.92	1.06	0.15	0.33	1.41	0.95	-73.20	9.99
	PLA-A	34.94	3.94	31.21	3.53	3.73	1.75	2.69	1.31	1.29	1.04	-55.05	8.90
	PLA-C	32.74	2.53	30.94	2.41	1.80	0.78	0.83	0.50	0.97	0.61	-68.88	6.81
	PLA-T	33.59	3.51	29.18	2.53	4.41	2.43	2.13	1.64	2.28	1.80	-51.88	11.68
Ti6Al4V	Control	37.90	4.00	29.40	1.70	8.60	3.60	4.50	0.70	4.00	3.30	-36.70	10.00
	PLA-A	34.37	2.08	31.25	1.56	3.12	1.37	0.19	0.16	12.78	2.11	-28.92	5.59
	PLA-C	35.62	1.65	30.60	1.11	5.02	1.22	0.56	0.26	11.25	1.63	-30.67	4.35
	PLA-T	32.66	1.87	32.06	0.88	0.60	1.65	0.01	0.03	14.80	4.73	-25.90	12.29
